# Computational reverse chemical ecology: Virtual screening and predicting behaviorally active semiochemicals for *Bactrocera dorsalis*

**DOI:** 10.1186/1471-2164-15-209

**Published:** 2014-03-19

**Authors:** Kamala Jayanthi P D, Vivek Kempraj, Ravindra M Aurade, Tapas Kumar Roy, Shivashankara K S, Abraham Verghese

**Affiliations:** 1 National Fellow Lab, Division of Entomology and Nematology, Indian Institute of Horticultural Research, Bangalore, India; 2 Division of Plant Physiology and Biochemistry, Indian Institute of Horticultural Research, Bangalore, India

## Abstract

**Background:**

Semiochemical is a generic term used for a chemical substance that influences the behaviour of an organism. It is a common term used in the field of chemical ecology to encompass pheromones, allomones, kairomones, attractants and repellents. Insects have mastered the art of using semiochemicals as communication signals and rely on them to find mates, host or habitat. This dependency of insects on semiochemicals has allowed chemical ecologists to develop environment friendly pest management strategies. However, discovering semiochemicals is a laborious process that involves a plethora of behavioural and analytical techniques, making it expansively time consuming. Recently, reverse chemical ecology approach using odorant binding proteins (OBPs) as target for elucidating behaviourally active compounds is gaining eminence. In this scenario, we describe a “computational reverse chemical ecology” approach for rapid screening of potential semiochemicals.

**Results:**

We illustrate the high prediction accuracy of our computational method. We screened 25 semiochemicals for their binding potential to a GOBP of *B. dorsalis* using molecular docking (*in silico*) and molecular dynamics. Parallely, compounds were subjected to fluorescent quenching assays (Experimental). The correlation between *in silico* and experimental data were significant (*r*^2^ = 0.9408; *P* < 0.0001). Further, predicted compounds were subjected to behavioral bioassays and were found to be highly attractive to insects.

**Conclusions:**

The present study provides a unique methodology for rapid screening and predicting behaviorally active semiochemicals. This methodology may be developed as a viable approach for prospecting active semiochemicals for pest control, which otherwise is a laborious process.

## Background

Olfaction studies have experienced an upsurge with respect to chemical ecology and neuroethology of insects [1]. This was made possible through the discovery of proteins related to olfaction [2]. Olfaction is achieved through two low-molecular weight (10-20 kDa) proteins, odorant binding proteins (OBPs) and odorant receptors (ORs) [3,4]. OBPs are the first proteins to recognize and bind to odor molecules in the long cascade of olfactory signal transduction [5-7]. OBPs interact with odors that enter through tiny pores present on the insect’s antenna forming an OBP-Odor complex. The complex transports odor molecules to ORs thereby starting the signal transduction cascade leading to behavioural outputs [8-10]. Although ORs recognize odors even in the absence of OBPs [11], high concentration of OBPs in the sensillar lymph in insects elicit questions of their physiological role. It is suggested that OBPs are involved in olfactory response, concluding that the specificity resides in the OBPs rather than in the odor molecules [12]. This contradicts the results obtained in studies using olfactory receptors expressed in heterologous systems [13,14]. It seems logical for OBPs to have a role in insect olfaction, helping insect in perceiving specific odors in this malodorous world where insects forage, mate, oviposit and discriminate between species through specific odor molecules. From earlier studies it has become clear that OBPs of insects have relevance in olfaction, acting as a liaison between the external environment and behavioural output [15]. Postulating against the specificity of OBPs is their low diversity in species and their broad binding spectrum to molecules of different chemical structures [16,17]. There are strong evidences that OBPs are involved in odorant discrimination, receptor sensitivity and specificity [18-23]. With such fortified evidence on the involvement of OBP in insect olfaction, it is reasonable for OBPs to serve as a molecular target in identifying potential behaviourally active compounds [24-26].

'Reverse chemical ecology’ [26,27] is a new concept for screening of attractants based on the binding ability of OBPs to test compounds rather than going through series of behavioural bioassays. This approach involves the study of the binding potential between a characterized OBP (protein) and an odor molecule (ligands) which can be simulated using computers. Here we describe a “computational reverse chemical ecology” approach involving a high performance drug discovery method to predict behaviourally active compounds for *B. dorsalis*. The compounds were also subjected to tryptophan quenching (binding assay) and behavioural assays to prove the efficiency of our approach. Computational methods may accelerate the screening process, thus, limiting our focus to small number of potential compounds that may be used in pest management.

## Results

### Odorant-binding protein and 3D model prediction

**Figure 1:**
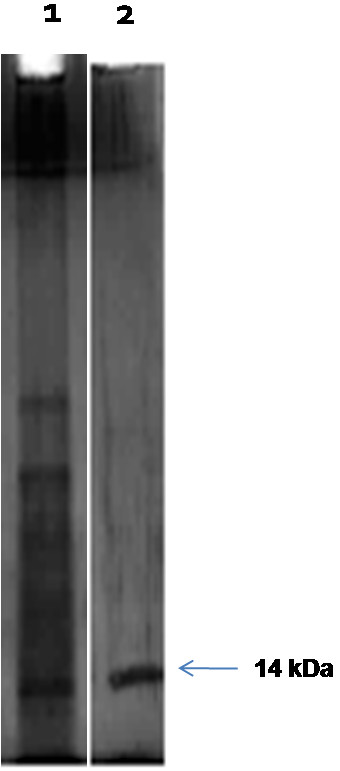
**SDS-PAGE analysis of antennal protein of *B. dorsalis.*** Silver stained SDS-PAGE (8% gel) showing Lane 1: Whole antennal protein and Lane 2: Purified OBP of *Bactrocera dorsalis*. The arrow shows an approx. 14 kDa OBP.

**Figure 2:**
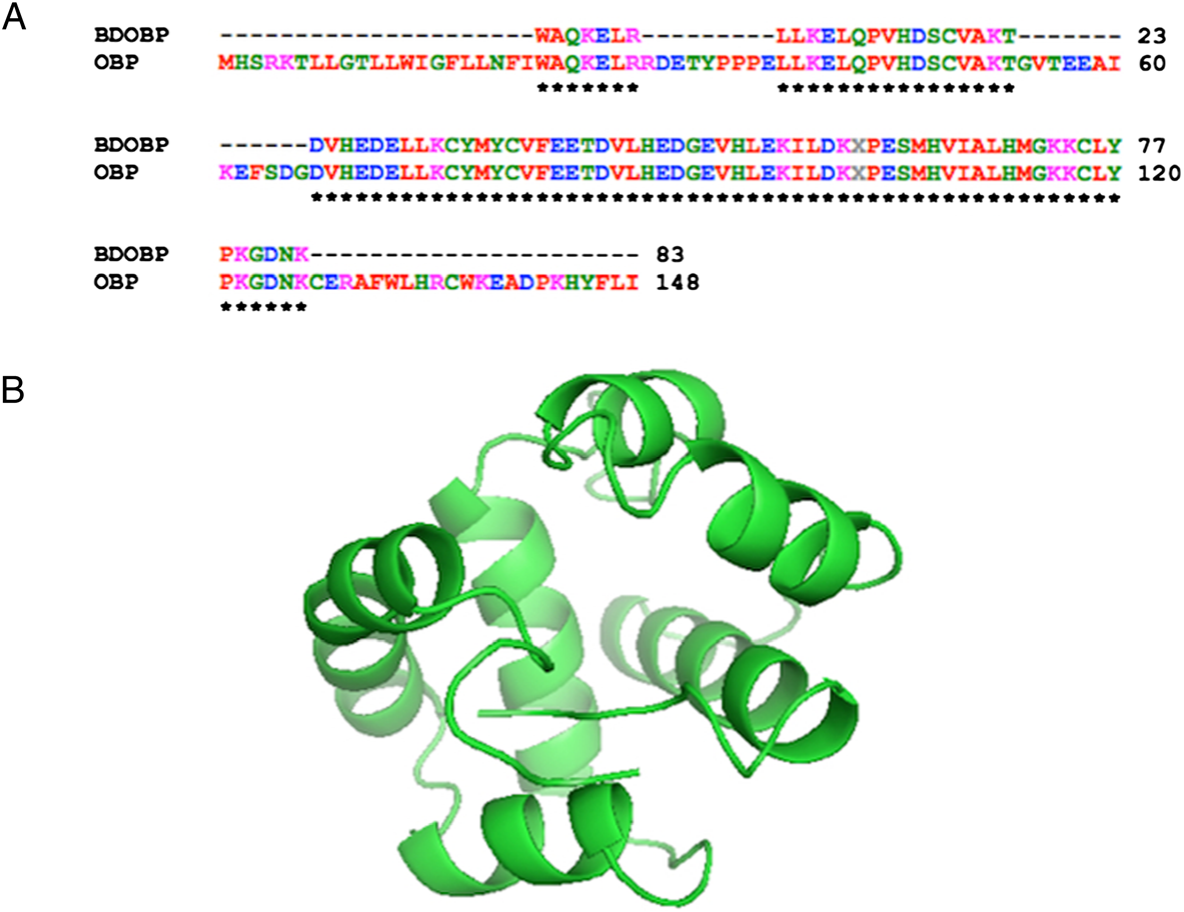
**Structural modeling of OBP of *B. dorsalis. *A** - sequence alignment between isolated OBP (BDOBP) and OBP of *B. dorsalis* in GenBank. Identical residues are highlighted with star below the letters. **B** - cartoon representation of OBP that was modelled using Phyre 2 protein threading software.

SDS-PAGE proved that the OBP was approximately 14 kDa in size and the isolated OBP was pure as evident by a single band in the gel (Figure 1) and demonstrated that the purified protein can be used for further investigation. The purified protein band was subjected to MALDI-TOF-MS and the partial sequence was blasted with submitted OBPs of *B. dorsalis* (Figure 2A). The BLAST results showed 100% sequence match with a previously isolated and characterized OBP of *B. dorsalis* (GenBank ID: ACB56577.1). Therefore, this previously sequenced protein was used for 3D model prediction. The Profile 3D score of the selected model was 48.34 and exceeded the minimum requirement value of 26.85. The predicted model consists of 6 α-helices that are located between 47-65 (α1), 72-85 (α2), 96-102 (α3), 105-118 (α4), 126-140 (α5) and 142-146 (α6). There also existed 3 pair of disulphide bridges that may play a role in stabilizing the structure (Figure 2B).

### Molecular docking, prediction and bioassay of behaviorally active compounds

A 3D structure of the isolated OBP with the highest score was selected and used in docking studies. We used an online molecular docking tool “Docking Server”. The 3D structures of protein and selected semiochemicals were loaded to the server. The results were processed and arranged for prediction. Thermodynamically, a ligand binds tightly to the active site of the protein when the free binding energy is low. Therefore, compounds with lower free binding energy were predicted to be behaviourally active. To aid in our prediction process compounds showing free binding energy less than -4.00 were considered behaviourally active and compounds with free binding energy more than -4.00 were used for comparison. First, we conducted a tryptophan quenching assay to find the binding potential of the selected compounds. Second, we conducted a behavioural assay to validate if the predicted compounds were behaviourally active or not. Tryptophan quenching was carried out with the isolated OBP and predicted compounds at concentrations ranging from 0 – 5000 nM. Kd value was estimated by fitting the fluorescence quenching data to an equation describing a single binding site present as a default in Prism Graph Pad version 5.01 for OS X. Percent quenching was determined and the graphs for all test compounds is shown in Additional file 1: Figure S1. The predicted compounds showed high quenching as evident by the Kd values. Kd values ranged from 600 – 6000 nM. The Trp fluorescence quenching spectrums of OBP with test compounds are shown in Additional file 2: Figure S2. The results are interesting because the compounds we predicted behaviourally active had tighter binding as evident by quenching and lower Kd values. Computer simulations or *in-vitro* binding assay of OBPs may not be an exact measure of the behavioural activity of an insect; however it may be relevant to the functional characterization of an OBP. Therefore, behavioural assays are needed to ascertain the nature (attractant or repellent) of the predicted compounds.

**Figure 3:**
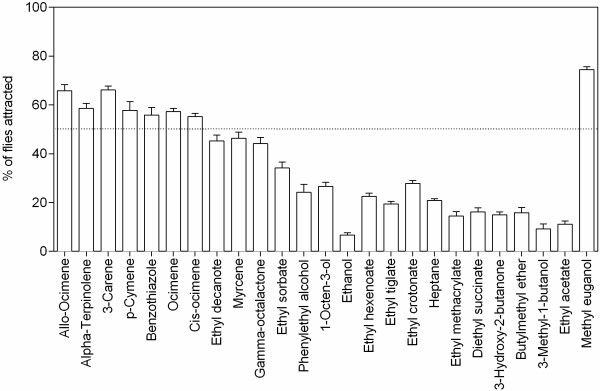
**Attraction efficiency of 25 kairomone compounds to *B. dorsalis*.** The test zone contained cellulose disc with 10 uL of individual kairomone and the control zone contained hexane. The mean number of insects in traps (respondents) and insects outside the trap (non-respondents) was used to determine a unified estimator, attraction index (AI). The % attraction was the mean of 9 individual experiments.

Behavioural assays were conducted to find the activity of predicted compounds. Using the behavioural assay data, a unified estimator, attraction Index (AI) was calculated (see Method for formula). From the data we found that methyl eugenol that was predicted as highly behaviourally active by its free binding energy showed the highest attraction 74.4% (Free binding energy = -5.63; AI = 0.50) and the lowest attraction was exhibited by ethanol with 6.67% (Free binding energy = -2.43; AI = -0.84) of flies attracted towards them (see Figure 3).

**Figure 4:**
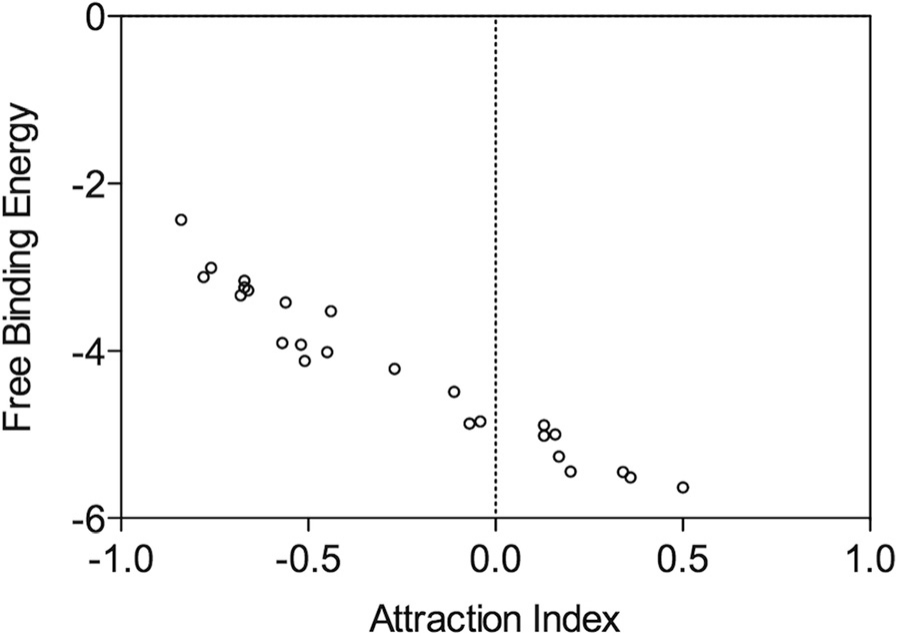
Correlation between attraction index and free binding energy.

**Figure 5:**
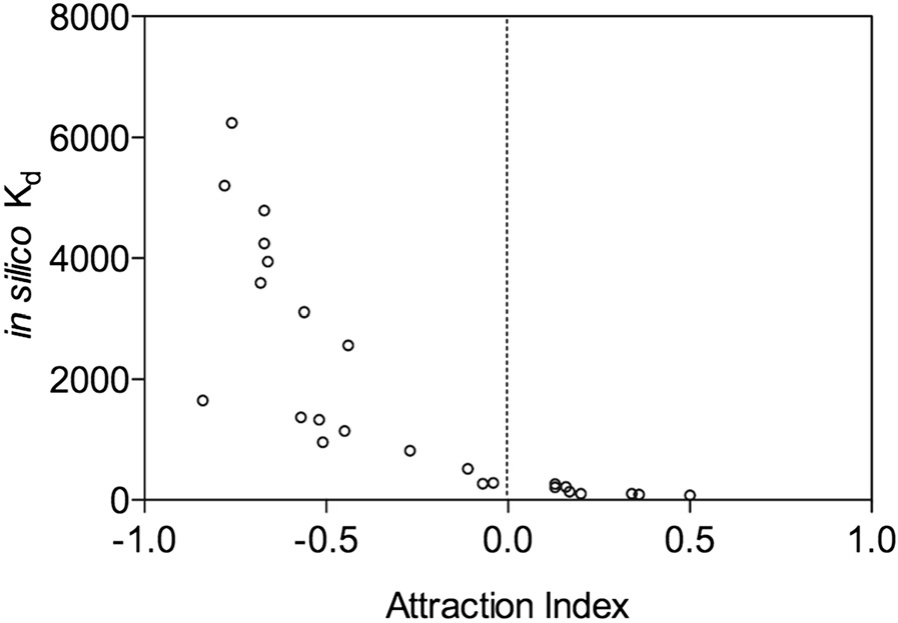
**Correlation between attraction index and *in silico *** **K**_**d**_**.**

**Figure 6:**
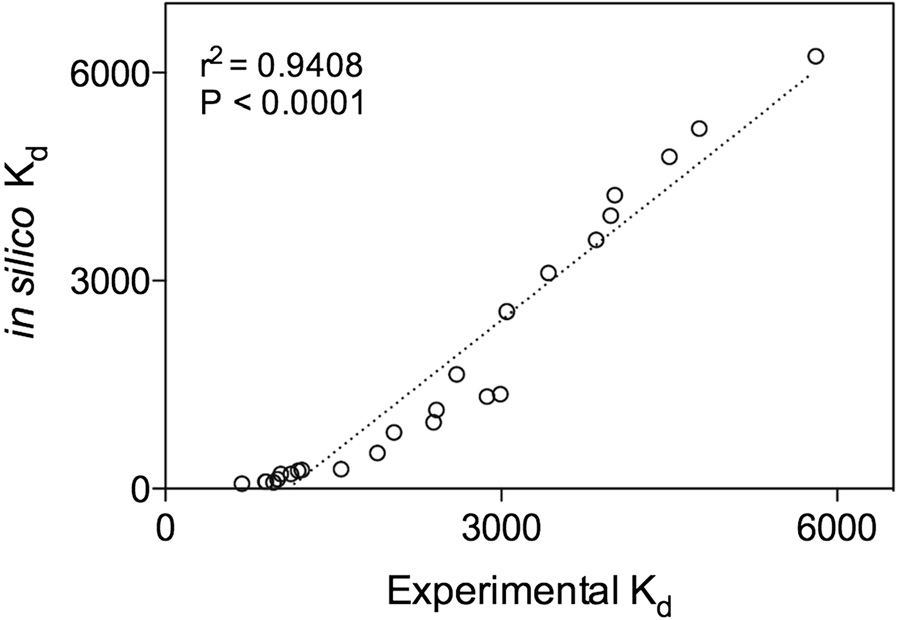
**Correlation between experimental K**_**d **_**and in silico K**_**d**_**.**

Statistical validation of computational and behavioural assays is crucial in such studies. As Kd and free binding energy are dependent, considering both for validation of the method is not sensible. Analysis carried out to standardize the dependable scoring functions for estimating the semiochemical efficiency showed significant correlation for both *in silico* Kd (Pearson r = -0.7974; *P* < 0.0001) and free binding energy (Pearson r = -0.9728; *P* < 0.0001) to AI (Figures 4 and 5). Regression analysis showed that the scoring function 'free binding energy’ (*F* = 90.41; *P* < 0.001; *r*^2^ = 0.9464) to be the best variable to predict behaviourally active compounds. Therefore, free binding energy was used as a dependable and robust scoring function. Then, correlation between *in silico* Kd and Experimental Kd was significantly positive with *r*^2^ = 0.9408 (*P* < 0.0001) and demonstrated that *in silico* data could be used for further studies (Figure 6).

## Discussion

Constant co-evolution between phytophagous insects and their host plants suggests that insects use chemical cues of their hosts to locate them. The tephritid fruit fly, *B. dorsalis*, uses a range of commercial fruit crops as hosts and cause huge losses to farmers in tropical and sub-tropical countries. The management option for this pest is mainly concentrated on males, thereby ignoring females that are otherwise highly the main cause of damage. Identifying potential attractants will definitely help us in narrowing down our focus on few potential compounds that may be attractive to females.

Recently, OBPs have shown to be required for the functioning of the olfactory system of insects [28-30] and has been used as a target protein in discovering attractants [26,27]. General odorant binding proteins (GOBPs) have a rather broad ligand-binding ability, in contrast to specificity. Therefore, using a consecutively produced general OBP as a target protein to a plethora of host volatiles may help us discover behaviourally active compounds. Although our method seems intriguing, it could not distinguish between repellents or attractants. Therefore, the method has been concluded with behavioural assays to verify the nature of the predicted compounds. Our method is a simple *in silico* approach for predicting and narrowing down to a set of behaviourally active compounds thereby reducing research cost and time.

## Conclusions

The results of the present study shows that our methodology used in this study is able to predict behaviourally active compounds accurately. Our unique approach helps us screen large number of compounds and predict their efficacy. It may be developed as a viable approach in discovering behaviourally active semiochemicals for integrated pest management strategies that otherwise is laborious and a costly affair. We anticipate that further improvements in the area of computational biology and proteomics may increase the efficiency of our method and short comes. Further the predicted compounds are to be validated in the field in case they are to be used for pest control.

## Methods

### Chemicals

All semiochemicals used were 99% pure and were purchased from Sigma-Aldrich, USA. Other chemicals were of analytical grade.

### Insects

The mango fruit fly, *B. dorsalis*, was reared on banana in the laboratory [31]. Fruits were exposed for 24 h to fruit fly cultures for oviposition. The oviposited fruits were kept in a plastic box containing fine sterilized sand. The sand was sieved after 10 days to aid in collection of pupa. The pupae were kept in cages for adults to emerge and were maintained at optimum conditions of 27 ± 2°C, 75% RH and 12:12 h dark light cycle. The emerged adults were fed with 10% honey solution and yeast extract *ad labium*. Antennae from gravid females (15 days old) were excised after emergence and stored at -20°C until use.

### Extraction, purification and characterization of OBP

A consecutively produced OBP was selected for the study and was extracted from the antenna of gravid, 15-day-old female *B. dorsalis*. Antennas collected were homogenized in cold 50 mM Tris-HCl buffer, pH 7.4. The homogenate was centrifuged at 10,000 rpm for 10 min at 4°C. The protein concentration was confirmed using Bradford method [32]. OBP was precipitated using 60% ammonium sulphate and dialyzed against 50 mM Tris-HCl buffer, pH 7.4. Most of the OBPs are in the range of 10-15 kDa, therefore, we used a 15-kDa cut off membrane to purify our protein. Further, a single band with molecular weight of approximately 14 kDa was purified by preparative SDS-PAGE. The corresponding band was electro-eluted for further analysis. The protein purity was analyzed by SDS-PAGE [33]. The band containing purified protein was cut and digested with trypsin under sterile condition and submitted to the Molecular Biophysics Unit, IISc, Bangalore for *De-novo* sequencing of the OBP using MALDI-TOF/LC-MS. The MALDI-MS spectra were searched for entries among the database. The partial sequence was compared with those of the proteins from MASCOT or BLAST similarity search to obtain homology.

### Selection of compounds for docking studies

Twenty-five volatile compounds that are identified in the headspace volatiles of the favoured host (Mango cv. Alphonso and Chausa) of *B. dorsalis* were selected for our study.

### Sequence retrieval and 3D modeling of OBP

During our homology search an antenna OBP of *Bactrocera dorsalis* with homology to our isolated protein existed in the protein sequence database (GenBank ID: ACB56577.1) of the National Centre for Biotechnology Information. A thorough search for the three dimensional structure of the OBP in PDB (Protein Database: http://www.rcsb.org/pdb) ascertained the non-existence of 3D structure of this protein. Therefore, the complete OBP submitted in GenBank was used for construction of a 3D model for our *in-silico* docking studies. Three-dimensional modelling of the antennal OBP of *B. dorsalis* was developed using an online protein threading (PHYRE 2) and homology modelling (SWISS-MODEL) online programs. AgamOBP1, a female odorant binding protein from *Anopheles gambiae* (PDB code: 2ERB) [34], CquiOBP1, a female-dominant odorant binding protein from *Culex quinquefasciatus* (PDB code: 3OGN) [35,36] and AaegOBP1, a major female- enriched odorant-binding protein from *Aedes aegypti* (PDB code: 3K1E) [6] were suggested as templates by the program. Multiple sequence alignment of the OBPs was carried out using Clustal O (see Additional file 3). It was found that the proteins had a identity of 48.993%. Identical and similar positions were 73 and 36 respectively. Based on the identified structural templates and the corresponding sequences, several 3D models were constructed using Modeler module in Discovery Studio 2.0 (Accelrys Software Inc., USA). The unaligned residues were deleted and the proteins were refined. The profiles-3D method was used as a standard in evaluating the fitness between the sequences and the 3D models. The model with the highest score of profile-3D was optimized and considered for further processing. Multiple sequence alignment (MSA) of the protein sequence used in the study is provided (see Additional file 3).

### Molecular docking and molecular dynamics simulations

Molecular docking was carried out using “Docking server” [37]. All compounds that were proved attractive to *B. dorsalis* from previous studies were considered for docking studies. Three-dimensional structures of compounds were from NCBI. Scoring function, free energy of binding was considered in tagging the compounds active. Docking was carried out 10 times with the selected OBP and the ligands (semiochemicals). For each run, the 10 highest scoring docking poses were saved and were further processed for molecular dynamics simulations and the free binding energies were calculated as previously described [38]. Briefly, all simulations were performed using AMBER 8.0 to suit our system. The ff03 force field was used and the time step was set at 0.5 fs. The temperature was set and maintained to a constant 300 K or 27°C and MD simulations were preformed for 700 ps for equilibration. The MD simulation calculations and the estimation of free binding energies by the MM-PB/SA method were preformed simultaneously. We did not use the MDGRAPE-3 system [38] for our simulations; therefore the average time was around 16-18 h per simulation. However, using faster systems may help us speedup the process. For calculation of free binding energy, the production MD trajectory was recorded for the last period of 610 ps. This was done as our protein was modelled and may contain errors. The MM-PB/SA was employed for calculating the free energy of binding and the equation described by Okimoto et al [37] was used. Each free binding energy of a protein-ligand complex is the minimum energies from among the energies of multiple poses (atleast 10 poses). All docking and MD simulations were done at pH 7.

### Measurement of ligand-OBP binding affinity by tryptophan fluorescence quenching

Tryptophan (Trp) fluorescence quenching assay was carried using Varian Cary Eclipse Spectroflurometer as described previously [34]. Briefly, OBP (10 ug/ml) was titrated in 50 mM Tris- HCl buffer, pH 7.4 with increasing concentration of ligands, while quenching of Trp fluorescence was monitored at 307 nm following excitation at 280 nm. Slit width for excitation and emission was 5 and 10 nm, respectively. Kd and ∆F max values were noted following fitting of data to an equation describing binding to a single affinity site. Quenching percent (% Q) was calculated using the formula, % Q = (*Δ*F/F0 × 100) in relation to the initial value after addition of ligand at a given concentration [S]. F0 and ∆F were initial fluorescence intensity and fluorescence intensity after addition of ligand, respectively.

### Behavioural assay

Forty *B. dorsalis* gravid females were released into an assay cage measuring 30×30×30 cm and were allowed to acclimatize. A test zone was determined and outlined with a cellulose disc (5 cm diameter) in a small plastic fly trap. Semiochemical stocks of predicted attractants and non-attractants were prepared by dissolving a specific amount of the compounds in redistilled hexane to give a final concentration of 0.05 ppm. A known amount (10 uL) of the semiochemical was dispensed on to the test zone. The traps were allowed for the solvent to evaporate and placed in assay cages. The number of insects trapped in the test zone was counted after 24 h. The experiment was repeated 9 times. The mean number of insects in traps (respondents) and insects outside the trap (non-respondents) was used to determine a unified estimator, attraction index (AI), that was calculated using the formula: AI = (# mean respondents - # mean non - respondents)/(# mean respondents + # mean non - respondents), where the # mean respondents indicates the mean number of insects in the trap and # mean non-respondents indicates the number of insects that did not respond. All data related to in silico and in vitro studies are provided in Additional files 4 and 5.

## Competing interests

The authors declare that they have no competing interests.

## Supplementary Material

Additional file 1 **Fluorescence quenching curves of 25 ligands to OBP of *B. dorsalis.*** Percent quenching of tryptophan is shown in the graphs. Click here for file

Additional file 2 **Fluorescence quenching spectrum of 25 ligands to OBP of *B. dorsalis.*** OBP was titrated with increasing concentration of test compounds. The upper first peak (red) is the spectrum of OBP alone. The following spectrum or lines corresponds to the increasing concentrations of the individual test compounds titrated. The Trp fluorescence λex: 280 nm, λem: 307, Slit width 5 (λex) and 10 nm (λem) at 24°C. Data are means of three independent experiments. Click here for file

Additional file 3 Multiple sequence alignment of OBPs in this study. Click here for file

Additional file 4 ***In silico *** **analysis of 25 semiochemicals docked with OBP and the outputs by the “Docking Server”.** Click here for file

Additional file 5 **Consolidated data of behavioural, *in vitro *** **and *in silico *** **experiments.** Click here for file
