# Supplementary figures and images for: Computational reverse chemical ecology: Virtual screening and predicting behaviorally active semiochemicals for Bactrocera dorsalis
Source: BMC Genomics. 2014 Mar 19;15:209. doi: 10.1186/1471-2164-15-209 (PMC4003815; doi:10.1186/1471-2164-15-209)

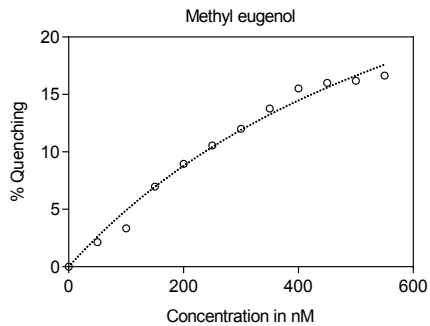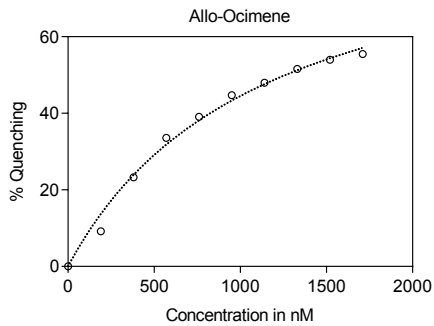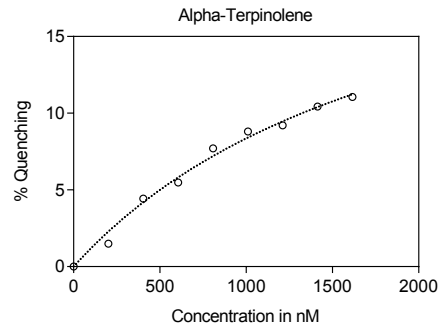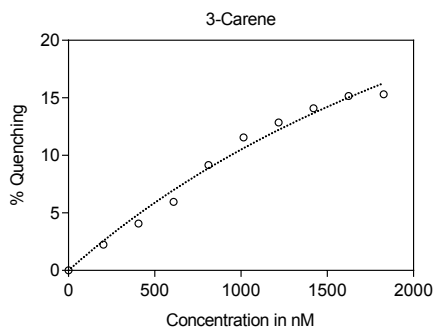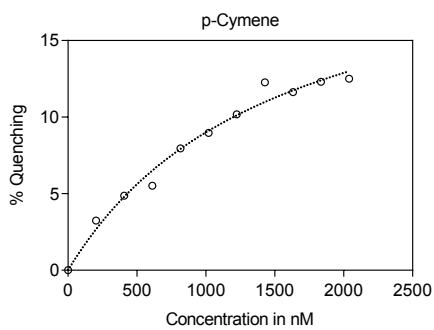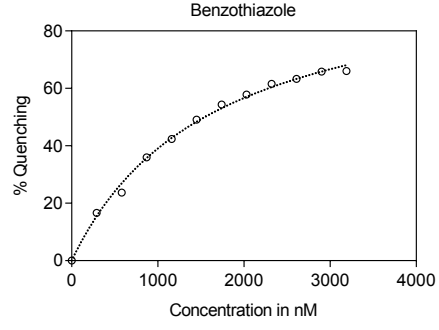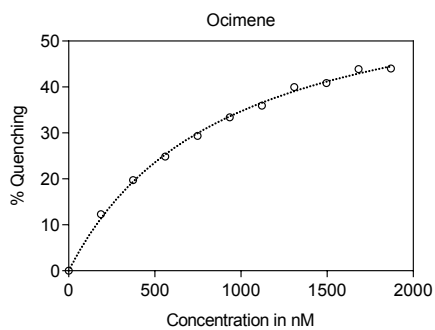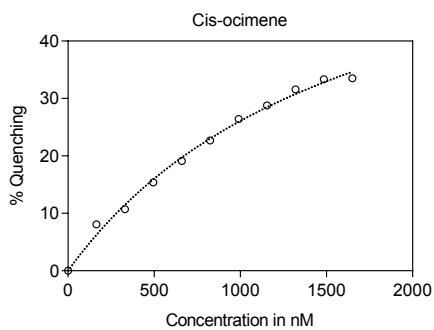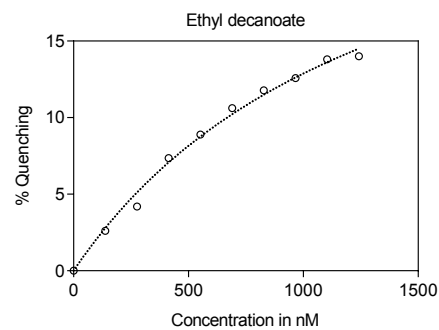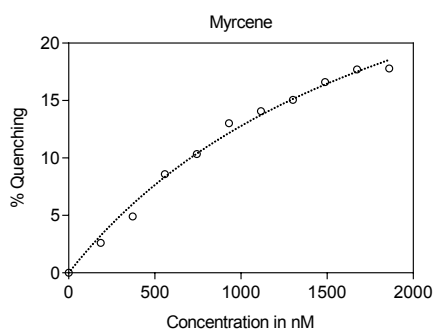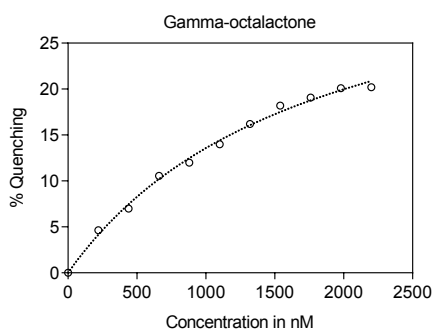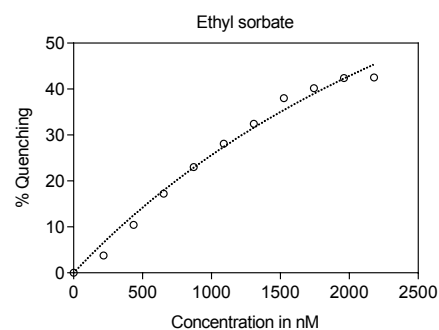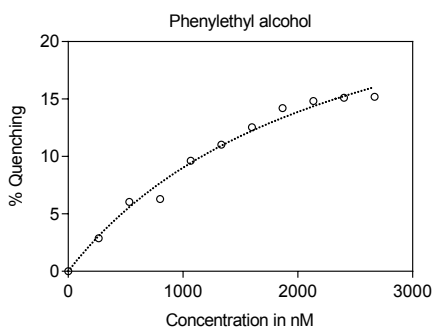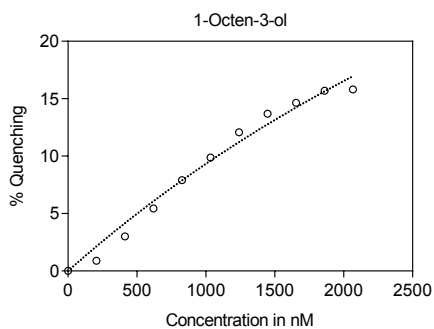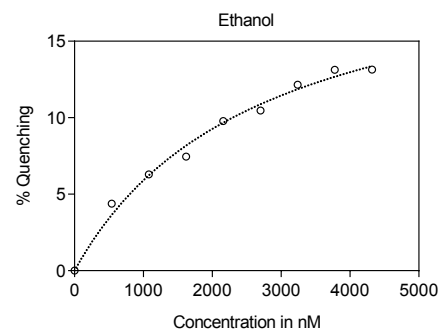

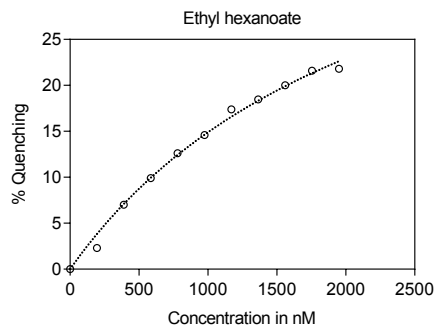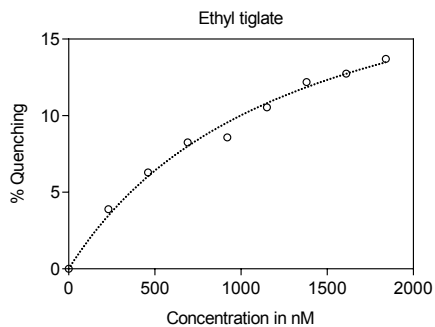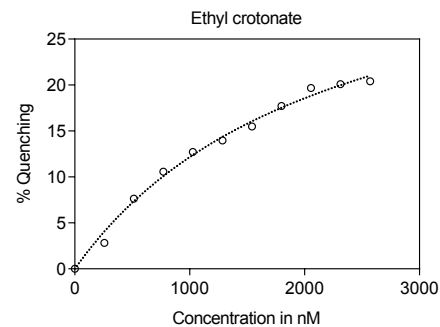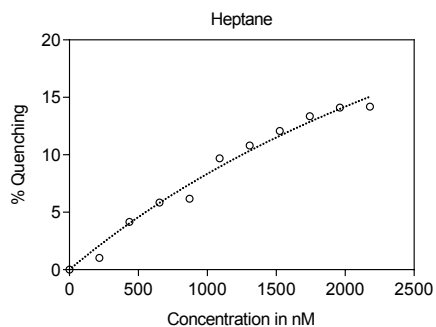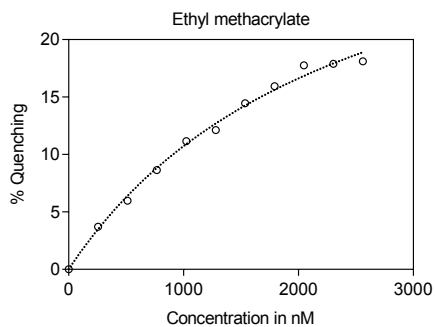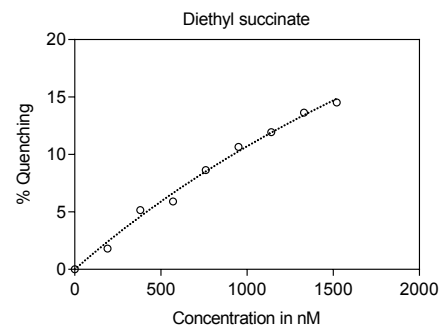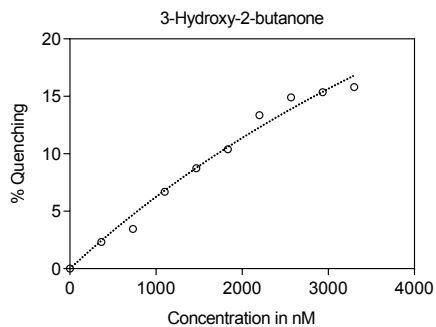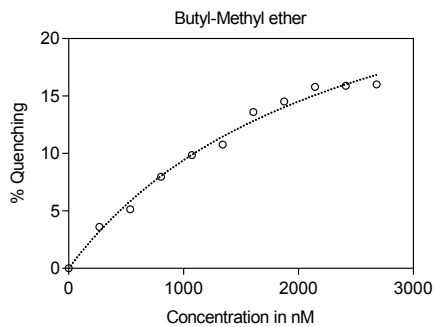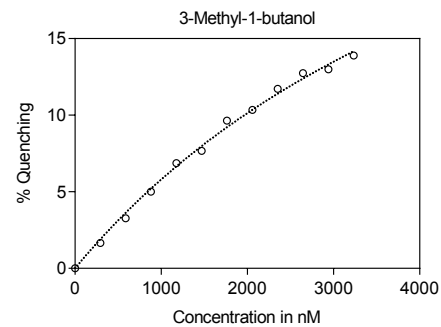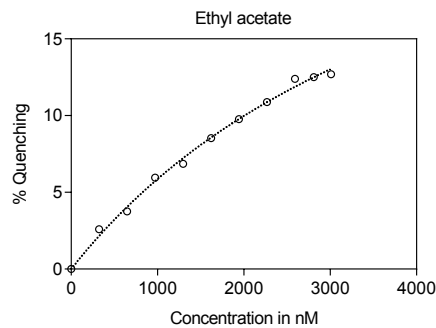

Supplement: Additional file 1 — Fluorescence quenching curves of 25 ligands to OBP of B. dorsalis. Percent quenching of tryptophan is shown in the graphs. [file 1471-2164-15-209-S1.pdf]

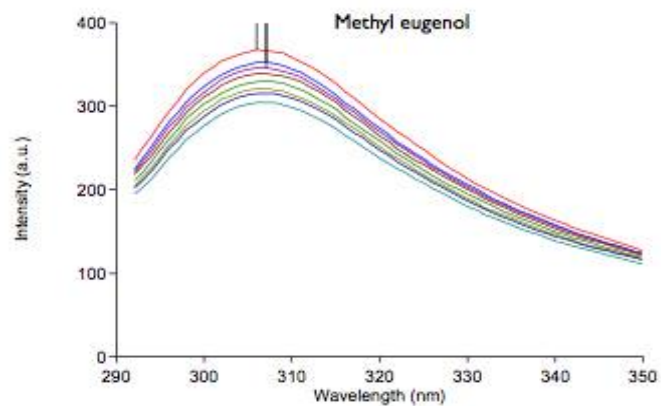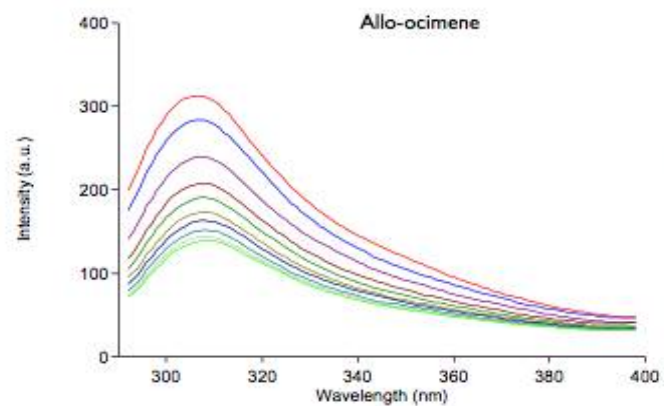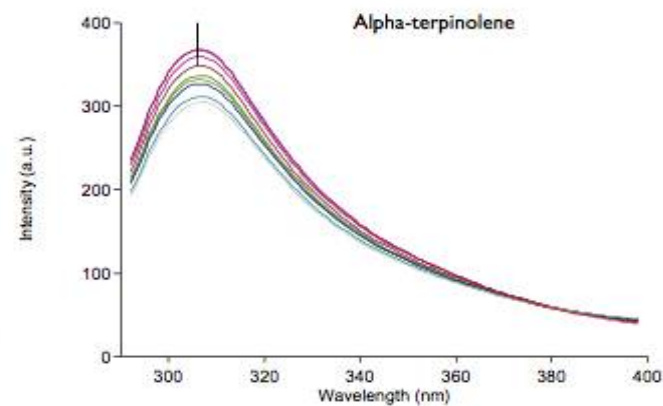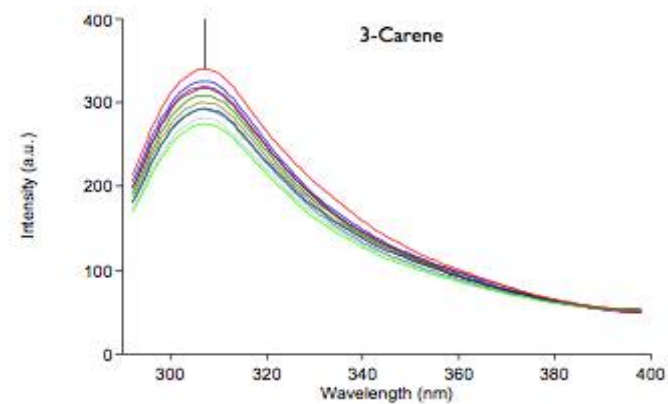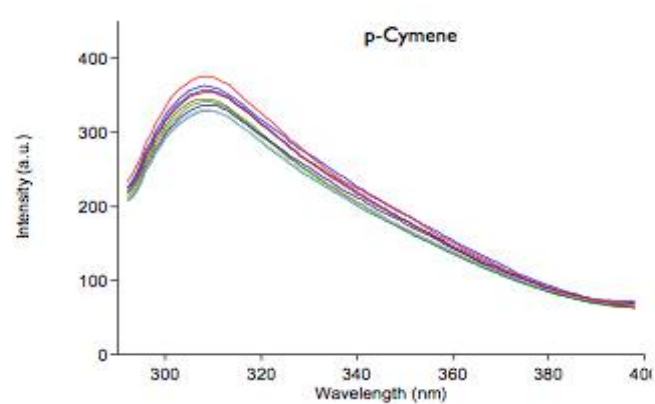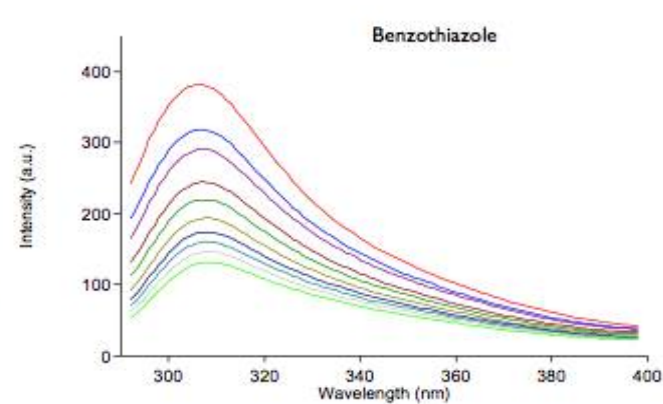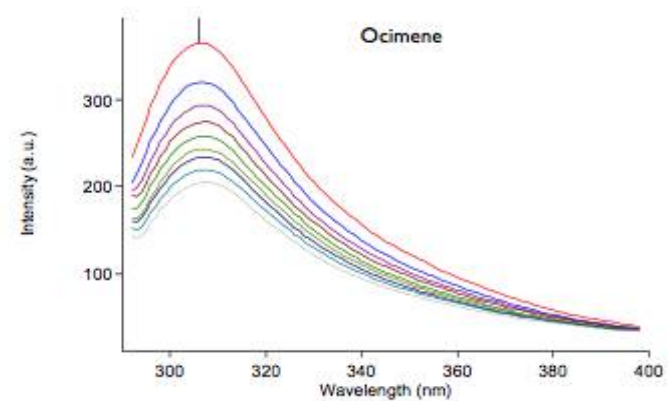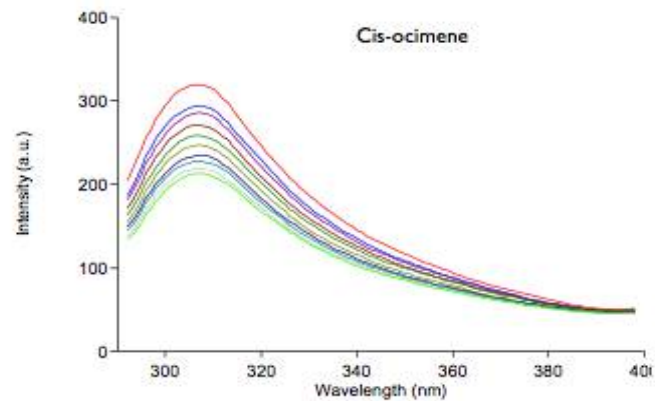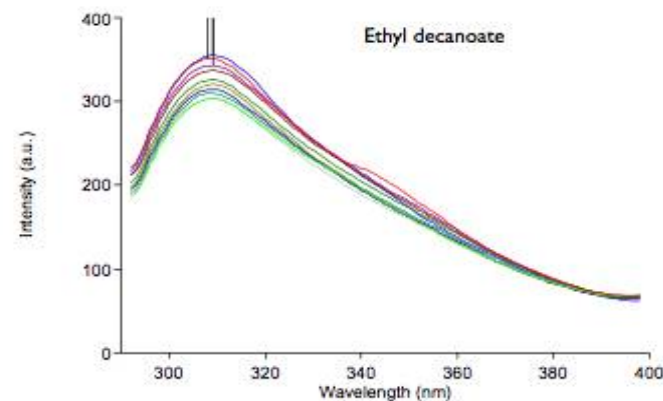

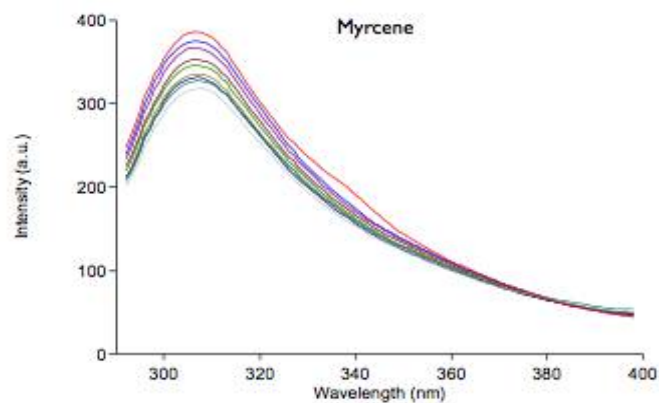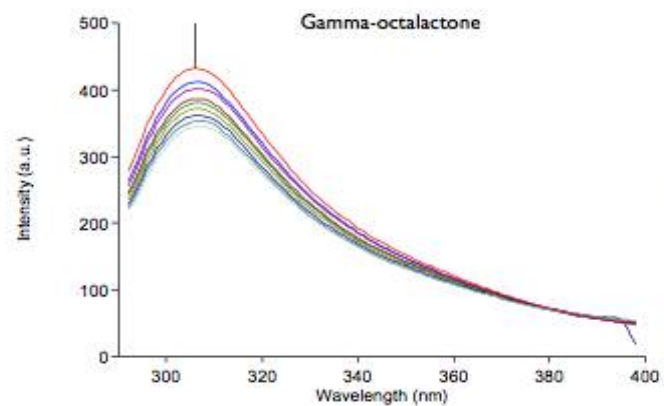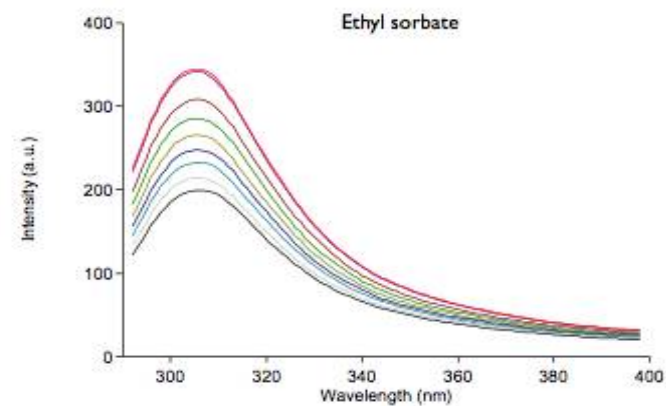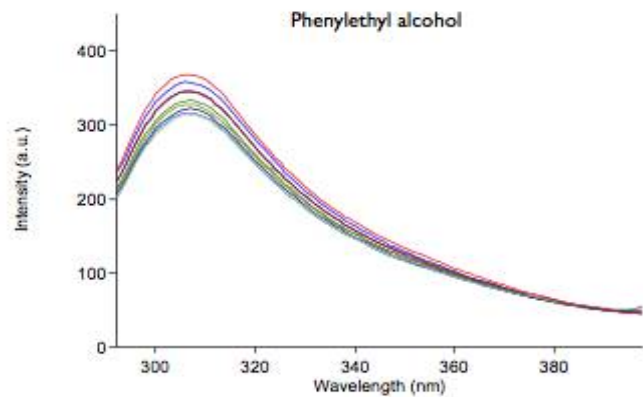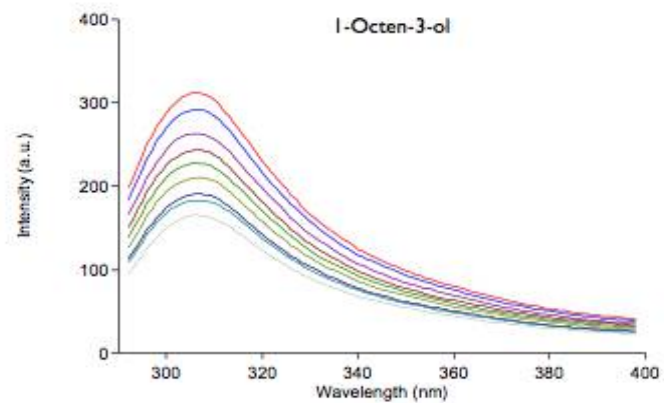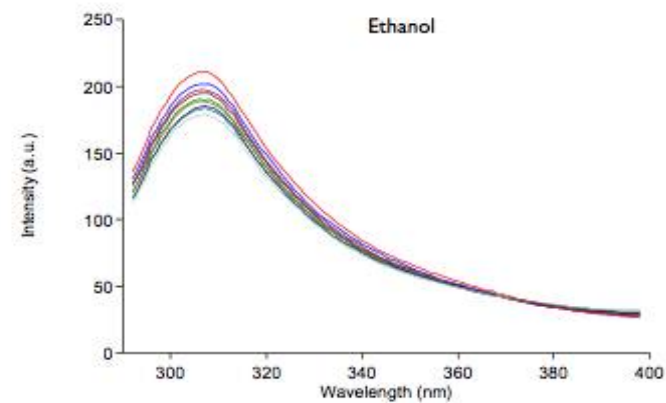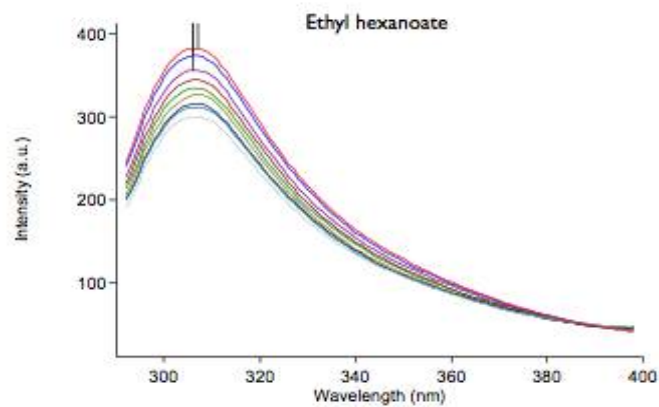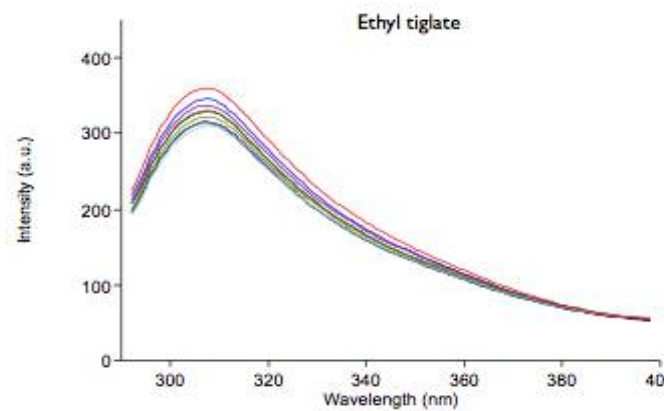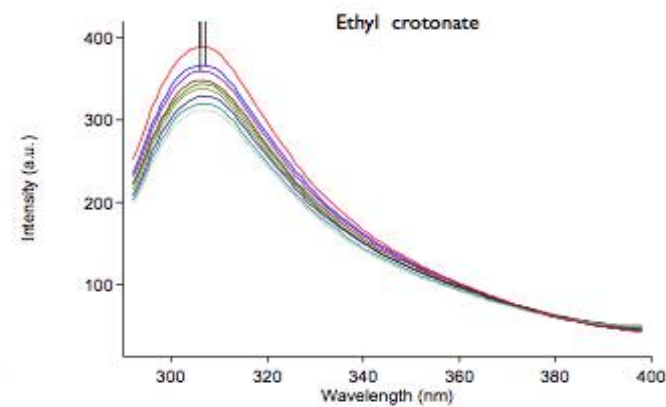

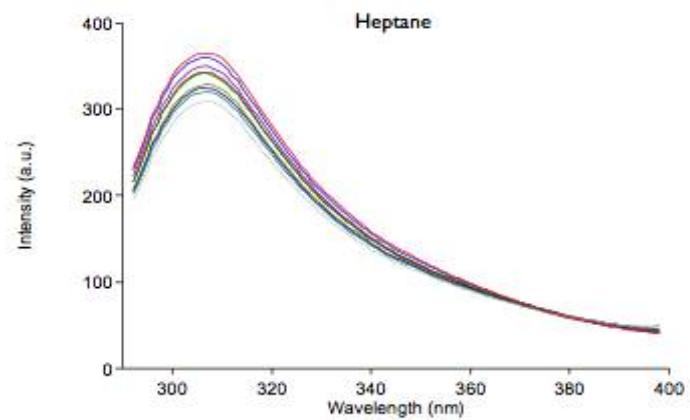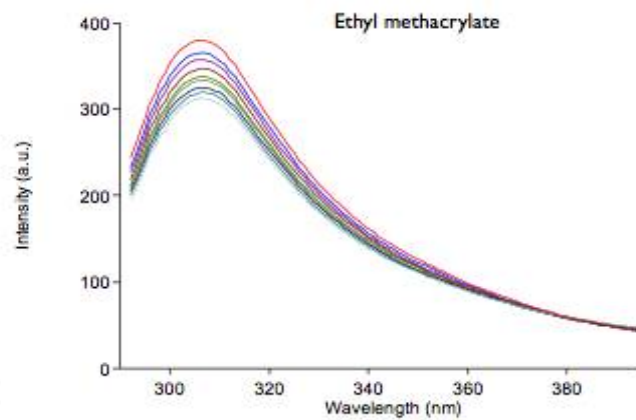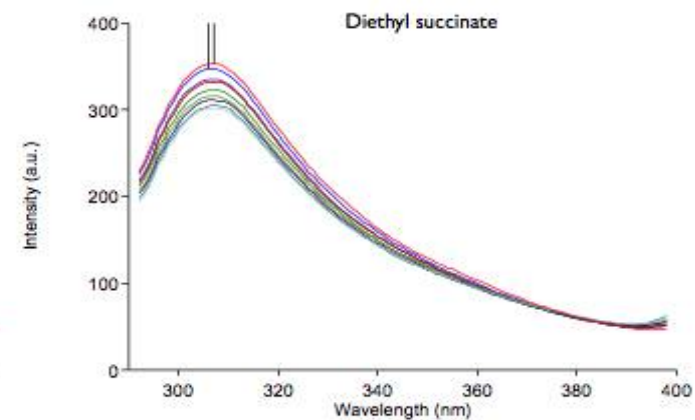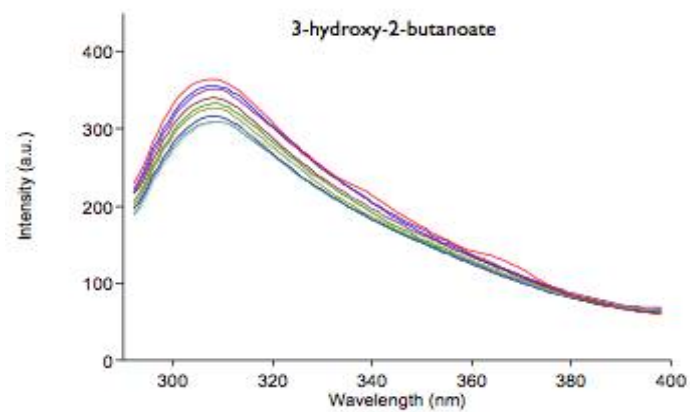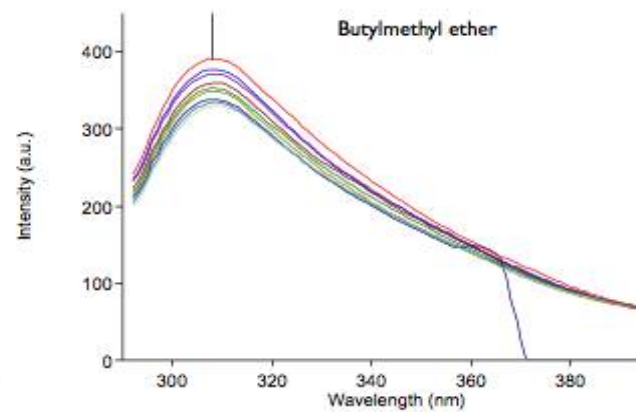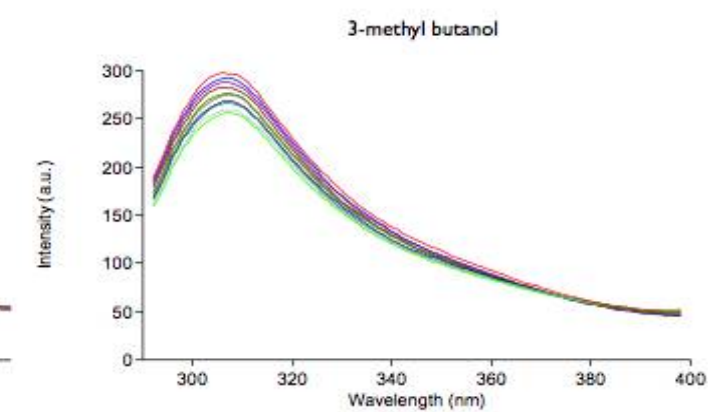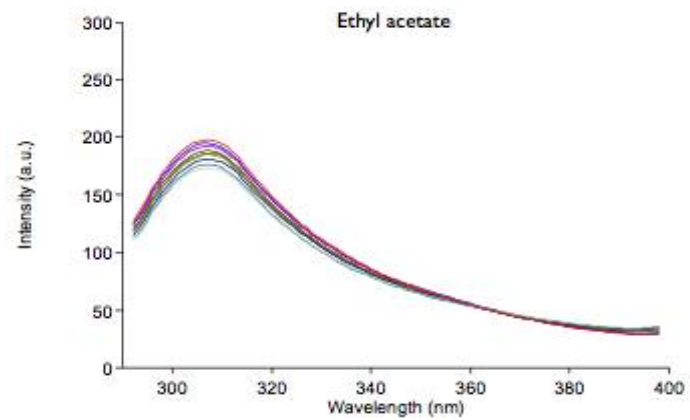

Supplement: Additional file 2 — Fluorescence quenching spectrum of 25 ligands to OBP of B. dorsalis. OBP was titrated with increasing concentration of test compounds. The upper first peak (red) is the spectrum of OBP alone. The following spectrum or lines corresponds to the increasing concentrations of the individual test compounds titrated. The Trp fluorescence λex: 280 nm, λem: 307, Slit width 5 (λex) and 10 nm (λem) at 24°C. Data are means of three independent experiments. [file 1471-2164-15-209-S2.pdf]
