# Supplementary material for: Computational reverse chemical ecology: Virtual screening and predicting behaviorally active semiochemicals for Bactrocera dorsalis
Source: BMC Genomics. 2014 Mar 19;15:209. doi: 10.1186/1471-2164-15-209 (PMC4003815; doi:10.1186/1471-2164-15-209)
Supplement: Additional file 3 — Multiple sequence alignment of OBPs in this study. [file 1471-2164-15-209-S3.pdf]

```
CLUSTAL O(1.2.0) multiple sequence alignment

Bactrocera MHSRKTLLGTLLWIG-FLLNFIWAQKELRRDETYPPPELLKELQPVHDSCVAKTGVTEEA 59
Anopheles  -----MKLVTFVFAALLCCSMTLGDTTPRRDAEYPPPELLEALKPLHDICLGKTGVTEEA 55
Culex      MAARCAKTLVLFSAVLGIAVVVLADVTPRRDAEYPPPELLEALKPLHDICAKKTGVTDEA 60
Aedes      ------MNGSVVFVLSALVSLSVGDVTPRRDAEYPPPEFLEAMKPLREICIKKTGVTEEA 54
                     ..        .  .:   ***  *****:*: ::*::: *  *****:**

Bactrocera IKEFSDGDVHEDELLKCYMYCVFEETDVLHEDGEVHLEKILDKXPESMHVIALHMGKKCL 119
Anopheles  IKKFSDEEIHEDEKLKCYMNCLFHEAKVVDDNGDVHLEKLHDSLPSSMHDIAMHMGKRCL 115
Culex      IIEFSDGKIHEDEKLKCYMNCLFHEAKVVDDNGDVHLEKLHDSLPNSMHDIAMHMGKRCL 120
Aedes      IIEFSDGKVHEDENLKCYMNCLFHEAKVVDDTGHVHLEKLHDALPDSMHDIALHMGKRCL 114
           * :*** .:**** ***** *:*.*:.*:.: *.*****: *  *.*** **:****:**

Bactrocera YPKGDNKCERAFWLHRCWKEADPKHYFLI 148
Anopheles  YPEGETLCDKAFWLHKCWKQSDPKHYFLV 144
Culex      YPEGENLCEKAFWLHKCWKQADPKHYFLV 149
Aedes      YPEGENLCEKAFWLHKCWKESDPKHYFL- 142
           **:*:. *::*****:***::*******
```
